# Comparison among *amoA* Primers Suited for Quantification and Diversity Analyses of Ammonia-Oxidizing Bacteria in Soil

**DOI:** 10.1264/jsme2.ME11230

**Published:** 2011-11-10

**Authors:** Yumi Shimomura, Sho Morimoto, Yuko Takada Hoshino, Yoshitaka Uchida, Hiroko Akiyama, Masahito Hayatsu

**Affiliations:** 1 National Institute for Agro-Environmental Sciences, 3–1–3, Kannondai, Tsukuba, Ibaraki 305–8604, Japan

**Keywords:** ammonia-oxidizing bacteria (AOB), *amoA* primer, real-time PCR, DGGE, primer degeneracy

## Abstract

Ammonia monooxygenase subunit A gene (*amoA*) is frequently used as a functional gene marker for diversity analysis of ammonia-oxidizing bacteria (AOB). To select a suitable *amoA* primer for real-time PCR and PCR-denaturing gradient gel electrophoresis (DGGE), three reverse primers (degenerate primer *amoA*-2R; non-degenerate primers *amoA*-2R-GG and *amoA*-2IR) were examined. No significant differences were observed among the three primers in terms of quantitative values of *amoA* from environmental samples using real-time PCR. We found that PCR-DGGE analysis with the *amoA*-2IR primer gave the best results in this studied soil. These results indicate that *amoA*-2IR is a suitable primer for community analysis of AOB in the environment.

Chemolithoautotrophic ammonia-oxidizing bacteria(AOB) play an important role in the global cycling of nitrogen (11). AOB convert ammonia (NH_3_) to nitrite (NO_2_^−^) through nitrification, which consists of the following two steps: oxidation of NH_3_ to hydroxylamine (NH_2_OH) by ammonia monooxygenase (AMO) (14) and conversion of NH_2_OH to NO_2_^−^ by hydroxylamine oxidoreductase (13, 15). During nitrification, nitrous oxide (N_2_O), a major greenhouse gas emitted from agricultural fields, is produced by AOB via two processes (33); chemical decomposition of intermediates such as nitrosyl hydride produced during oxidation of NH_2_OH to NO_2_^−^(16) and nitrifier denitrification (24, 27), in which N_2_O is produced during reduction of NO_2_^−^(24, 27) with NH_2_OH as an electron donor (25). Fertilization of agricultural fields increases N_2_O emissions (3); however, the extent of AOB contribution and the species involved in N_2_O emissions in fields are not yet known because it is difficult to isolate individual bacterial species from the environment (1). Therefore, community analysis using PCR-based methods is important to understand the ecology of AOB in the environment.

A gene for subunit A of AMO (*amoA*) is frequently used as the functional marker of AOB in PCR-based methods for diversity analysis of AOB (26). Rotthauwe *et al.*(26) constructed a primer set consisting of a single primer *amoA*-1F and a degenerate primer *amoA*-2R, having degeneracy at position 7 (K=G or T) and 9 (S=C or G) from the 5′ end (Table 1). The primer set amplified *amoA* fragments of *Nitrosomonas* and *Nitrosospira* belonging to the β subclass of *Proteobacteria*(26). Oved *et al.*(23) developed a PCR-denaturing gradient gel electrophoresis (DGGE) method using *amoA*-1F attached to the GC-clump at the 5′ end (*amoA*-1F-GC) (Table 1) and the *amoA*-2R primer set. Furthermore, they identified its usefulness for community analysis of AOB. The *amoA*-1F(*amoA*-1F-GC)/*amoA*-2R primer set has frequently been used for diversity analysis of AOB (4, 22, 23); however, degeneracy of the *amoA*-2R primer results in smears and multiple DGGE bands (7, 17). Therefore, several studies have evaluated the usefulness of non-degenerate primers that correspond to the same region as *amoA*-2R (7, 8, 17, 20).

In *amoA*-2R-GG (20) and *amoA*-2IR (7) primers, the degenerate regions of *amoA*-2R are replaced by guanines and inosines, respectively (Table 1). *amoA*-2R-GG and *amoA*-2IR reportedly exhibit single-band patterns in DGGE (6, 9). Furthermore, *amoA*-2R-GG produces DGGE patterns similar to those of *amoA*-2R (7, 8). Although *amoA*-2IR has the potential to detect more diverse *amoA* sequences than normal base primers, the primer has not been used in community analysis of AOB in the environment, including soil. In this study, three primers, *amoA*-2R, *amoA*-2R-GG, and *amoA*-2IR, were used to analyze the quantitative capability and detectability of *amoA* in soil samples by real-time PCR and PCR-DGGE.

Soil samples were collected from experimental lysimeter plots, wherein N_2_O fluxes were monitored using an automated chamber system (2, 21), at the National Institute for Agro-Environmental Sciences, Tsukuba, Ibaraki prefecture in April, 2010. Two types of soil, gray lowland and andosol, were used in this study. In Japan, these are the most common soil types in agricultural fields. Gray lowland soil and andosol account for 22% and 18%, respectively, of all agricultural fields, including paddy fields, upland crop fields, grassland, and orchards (31). In particular, andosol, which is volcanic-ash soil, occupies 41% of the total upland crop fields in Japan (31). Gray lowland soil has the following properties: moisture, 26% (w/w); NH_4_-N, 1.87 mg kg^−1^; NO_3_-N, 0.55 mg kg^−1^; pH (H_2_O), 6.4; total C, 17.3 g kg^−1^; total N, 1.5 g kg^−1^; exchangeable K, 1.21 cmol(+) kg^−1^; and available P, 113 mg kg^−1^. Andosol has the following properties: moisture, 28% (w/w); NH_4_-N, 1.72 mg kg^−1^; NO_3_-N, 2.21 mg kg^−1^; pH (H_2_O), 6.3; total C, 31.6 g kg^−1^; total N, 2.6 g kg^−1^; exchangeable K, 1.11 cmol(+) kg^−1^; and available P, 211 mg kg^−1^. Five soil cores (diameter, 3 cm; depth, 5 cm) from each site were collected, pooled, and sieved (2 mm). Total community DNA was extracted from soil in triplicate using the FastDNA spin kit for soil (Q-Biogene/MP Biomedicals, Solon, OH, USA) as described previously (19). During extraction of DNA from andosol, 16 mg skim milk was added to prevent DNA adsorption and improve recovery (19, 30).

Real-time PCR was performed to measure the amplification efficiency of the three primer sets shown in Table 1(12). Four types of *amoA* clones, including the complementary sequence of *amoA*-2R, were constructed as templates (Fig. S1). *Nitrosospira multiformis* ATCC 25196 *amoA* (Nmul_ A2765) was used for the construction of clones with the four primer sets shown in Table S1. The PCR reaction mixture compositions and thermocycling conditions are shown in Table S2. The PCR reactions were performed using the iCycler thermal cycler (Bio-Rad Laboratories, Hercules, CA, USA). Each amplicon was purified using the QIAquick PCR Purification kit (Qiagen, Valencia, CA, USA), inserted into a pGEM-T Easy vector (Promega, Madison, WI, USA), and transformed to *Escherichia coli* strain DH5α (Toyobo, Tokyo, Japan). Plasmid DNA was extracted using the QIAprep MiniPrep kit (Qiagen) and sequenced using the ABI PRISM 3100 Genetic Analyzer (Applied Biosystems/Life Technologies, Carlsbad, CA, USA) and the BigDye Terminator v3.1 DNA sequencing kit (Applied Biosystems). The plasmids were linearized using *Hinc*II restriction enzyme, purified, and diluted in distilled water. DNA concentrations were measured using a spectrophotometer (Nanodrop 1000; Thermo Scientific, Wilmington, DE, USA), and then *amoA* copy number in the solution was calculated based on the determined DNA concentration and the molecular weight of the linearized plasmid. Serial dilutions containing between 10^3^ and 10^7^*amoA* copies were prepared from the solution of the linearized plasmid. Real-time PCR was performed using the StepOne Real-Time PCR System (Applied Biosystems) by the SYBR Green I method. The PCR conditions are shown in Table S2. Standard curves based on serial dilutions of each constructed clone with the three primer sets (Table 1) were generated by plotting the threshold cycle for each standard calculated by StepOne software, ver. 2.1 (Applied Biosystems).

*amoA* copy numbers in the extracted soil DNA were measured in triplicate with each of the three primer sets shown in Table 1. The mixed clones were serially diluted as described above to obtain the standard curve. Tukey’s test was used to evaluate the significance of differences of *amoA* copy numbers obtained from each primer.

Primer detection sensitivity for *amoA* from soil DNA was examined by PCR-DGGE. PCR was performed with each of the three primer sets shown in Table 1 and the extracted soil DNA. The composition of PCR reaction mixtures and the thermal cycling conditions are shown in Table S2. To optimize the annealing temperatures, four annealing temperatures, from 52°C to 58°C in increments of 2°C, were used to examine changes in the band patterns (Table S2). All amplicons were purified and adjusted to 200 ng DNA per well and used for DGGE (*n*=2), which was performed using the DCode universal mutation detection system (Bio-Rad Laboratories). An 8% (w/v) polyacrylamide gel with a denaturant gradient ranging from 50% to 65% was prepared. The procedures of electrophoresis, gel staining, and gel imaging were the same as those described previously (9). The detected major DGGE bands were excised, re-amplified by PCR, and sequenced according to the methods described previously (8). All sequences obtained in this study were examined for chimeric sequences as described previously (18). The band sequences and obtained *amoA* sequences from the National Center for Biotechnology Information (NCBI) were translated into amino acid sequences and aligned using Clustal W (32). The neighbor-joining tree was constructed using MEGA, version 5.0 (Molecular Evolutionary Genetics Analysis [http://www.megasoftware.net/]) with the Jones–Taylor–Thornton amino acid substitution model.

The effect of primer mismatches on amplification efficiency was examined by real-time PCR using the constructed *amoA* clones, including the complementary sequences with *amoA*-2R. The standard curves obtained from each clone amplified by each of the three primer sets showed similar slope values (Table S3). The calculated amplification efficiencies of *amoA*-2R, *amoA*-2R-GG, and *amoA*-2IR ranged from 91.3% to 97.6% (mean: 95.0%), 93.0% to 96.7% (mean: 95.0%), and 91.1% to 94.7% (mean: 93.0%), respectively (Table S3). The mean efficiency of *amoA*-2IR was slightly lower than that of the *amoA*-2R and *amoA*-2R-GG primers. PCR biases derived from primer mismatches were not detected when complementary *amoA* sequences of *amoA*-2R were used as templates.

No significant differences were observed among the three primer sets in terms of quantitative values of *amoA* copy numbers in gray lowland soil (1.86–2.03×10^7^ copies [g dry soil]^−1^) and andosol (2.80–3.06×10^7^ copies [g soil]^−1^) (Fig. 1). Even though amplification efficiencies differed among the primers, each quantitative value was apparently corrected by each standard curve.

To optimize the annealing temperature for PCR-DGGE, four annealing temperatures (52, 54, 56, and 58°C) were examined. At 58°C, *amoA*-2R and *amoA*-2R-GG exhibited good *amoA* detectability in soil samples (Fig. S2); *amoA*-2IR produced more bands at 52°C than at 54°C or higher (Fig. S2). The band patterns of the soil samples observed under previously described annealing conditions are shown in Fig. 2. The patterns obtained from the three primer sets for gray lowland soil were similar, whereas the band migration distances varied with the primers. The distance variances were apparently caused by differences in the primer sequences. The band pattern obtained from andosol was more complex than that from gray lowland soil (Fig. 2). The band patterns of *amoA*-2R and *amoA*-2R-GG obtained from andosol were similar. In contrast, *amoA*-2IR produced a different band pattern (Fig. 2). The number of bands obtained from *amoA*-2IR was 14, and it was greater than 10 from *amoA*-2R and *amoA*-2R-GG.

All bands to which a number was assigned in Fig. 2 were sequenced and identified as *amoA* fragments. Chimeric sequences were not detected. In the band pattern obtained from gray lowland soil, all bands in each lane were found to exhibit identical nucleotide sequences (Fig. 3). No difference in detectable sequences was observed among the three primers from gray lowland soil samples. In the band pattern obtained from andosol, eight bands in each lane were found to exhibit identical nucleotide sequences (Fig. 3). Sequences retrieved from *amoA*-2R band 5 (Andosol; 2R-5) and 6 (2R-6) had the same nucleotide sequence (Fig. 3), which seemed to be caused by *amoA*-2R degeneracy. *amoA*-2IR detected not only almost all of the *amoA* sequences obtained by *amoA*-2R and *amoA*-2R-GG, but also several unique sequences (Fig. 3). The unique sequences were assigned to cluster 10 on the basis of translated amino acid sequences of *amoA*, and were classified using the previously described (5–7) nomenclature for *Nitrosospira amoA* (Fig. 3). Other researchers reported that cluster 10-related *amoA* sequences were difficult to detect from a mixed clone library of various *amoA* sequences using the *amoA*-1F/*amoA*-2R primer set (5, 7). *amoA*-2R and *amoA*-2R-GG each detected only one sequence (retrieved from gray lowland bands; 2R-5 and GG-5, respectively), which was the same nucleotide sequence with 2IR-5 assigned to cluster 10; however, their bands were not as clear as those from 2IR-5 (Fig. 2). These results indicate that DGGE analysis with *amoA*-2IR can reproduce the pattern of the *amoA* sequence in this studied soil environment with higher sensitivity than *amoA*-2R and *amoA*-2R-GG. Because inosine is able to form stable pairs with both cytosine and thymine (10), the amplification bias may not be caused by *amoA*-2IR.

Avrahami *et al.*(7) compared the detectability of *amoA* genes among the three primers used in this study with 8 *amoA* clones. They reported that detectability with *amoA*-2IR was less than that with *amoA*-2R and *amoA*-2R-GG. In this study, we used total soil DNA samples to evaluate the primers and found that *amoA*-2IR detected several unique sequences from andosol at low annealing temperature. The low annealing temperature may increase the *amoA* detectability of *amoA*-2IR (Fig. S2) because the *T*m value of *amoA*-2IR was 50.5°C, which was lower than that of *amoA*-2R (53.5°C) and *amoA*-2R-GG (54.4°C). Hornek *et al.*(17) reported that amoAr-I, which included inosine at position 13 from the 5′ end of *amoA*-2IR, detected *Nitrosomonas*-*amoA* sequences in environmental samples that were different from those detected by *amoA*-2R. In this study, *Nitrosomonas*-like *amoA* sequences were not detected from all samples. The population of *Nitrosomonas* species appeared to be lower than the detection limit in the soil samples. *Nitrosospira*-like *amoA* sequences have been shown to be more common and abundant than those from *Nitrosomonas* in soil environments (29).

In our study, *amoA*-2IR showed the same quantitative capability and superior detection sensitivity of *amoA* sequences as *amoA*-2R from soil samples compared with *amoA*-2R and *amoA*-2R-GG primers. Our results indicated that *amoA*-2IR is a suitable primer for community analysis of AOB in soil, as determined by both real-time PCR and PCR-DGGE. Our results also indicate that *amoA*-2IR is more useful for the analysis of AOB in andosol.

The sequences obtained in this study were deposited in the DNA Data Bank of Japan under accession numbers AB621399 to AB621419.

## Supplementary Material



## Figures and Tables

**Fig. 1:**
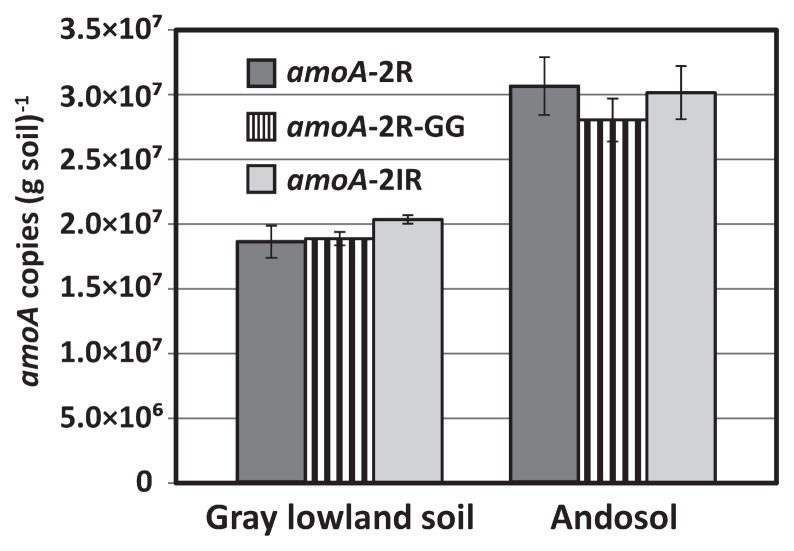
Abundance of *amoA* gene in gray lowland soil and andosol detected by each primer. Error bar designates SD for *n*=3.

**Fig. 2:**
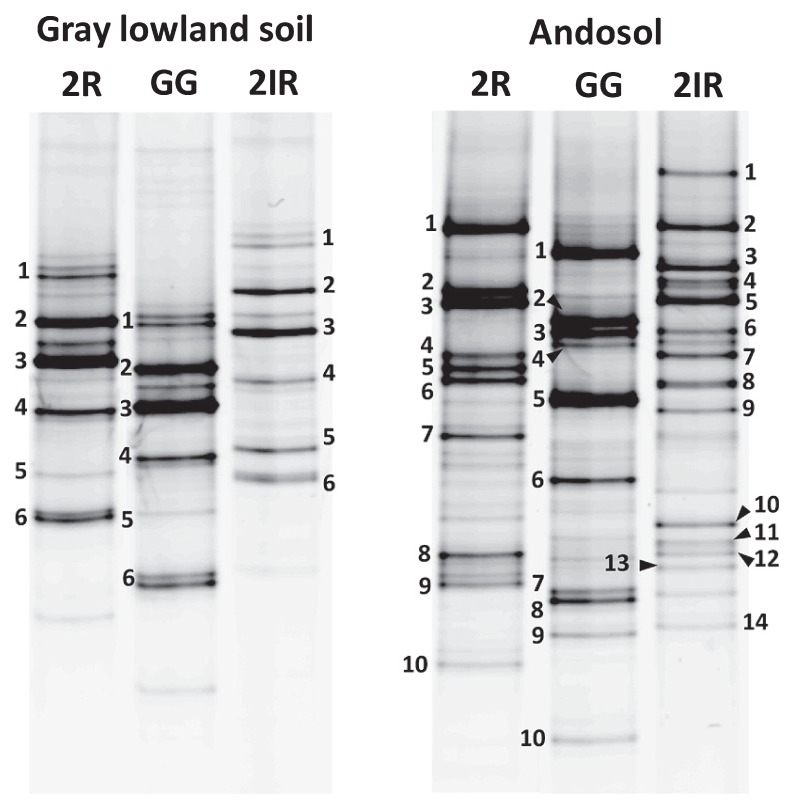
DGGE band patterns of *amoA* retrieved from gray lowland soil and andosol. Band patterns were produced at an annealing temperature of 58°C (*amoA*-2R and *amoA*-2R-GG) or 52°C (*amoA*-2IR) (Fig. S3). Numbered bands were sequenced. 2R, *amoA*-2R primer; GG, *amoA*-2R-GG primer; 2IR, *amoA*-2IR primer.

**Fig. 3:**
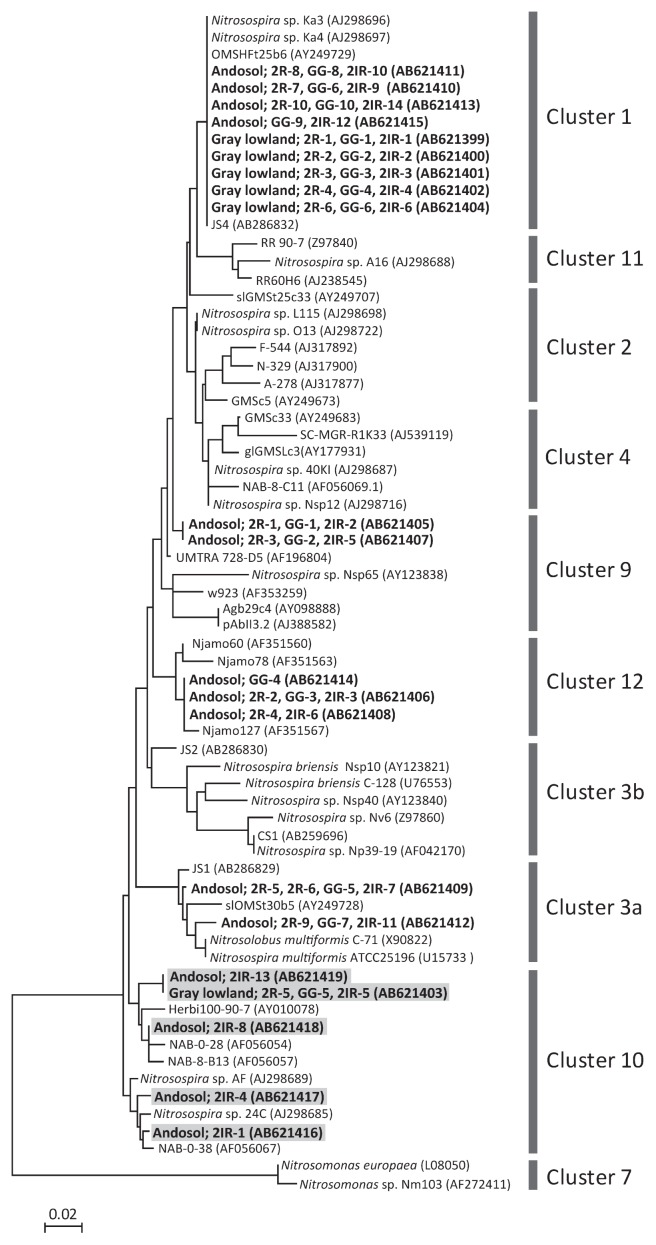
Phylogenetic tree based on partial *amoA* sequences (150 amino acids) retrieved from gray lowland soil and andosol in this study and obtained from NCBI. 2R, GG, and 2IR refer to the used primers: *amoA*-2R, *amoA*-2R-GG, and *amoA*-2IR, respectively. Subsequent numbers correspond to the DGGE bands shown in Fig. 2. Scale bar indicates two changes per 100 amino acid positions.

**Table 1:** PCR primers used in this study for the amplification of *amoA* gene fragments

Primer		Sequence (5′-3′)	*T*m (°C)	Reference
Forward	*amoA*-1F	GGGGTTTCTACTGGTGGT	46.5	(26)
	*amoA*-1F-GC*	**CGCCCGCCGCGCCCCGCGCCCGTCCCGCCGCCCCCGCC** **CG**GGGGTTTCTACTGGTGGT	84.2	(26, 28)
Reverse	*amoA*-2R	CCCCTC**K**G**S**AAAGCCTTCTTC (K=G or T, S=G or C)	53.5	(26)
	*amoA*-2R-GG	CCCCTC**G**G**G**AAAGCCTTCTTC	54.4	(20)
	*amoA*-2IR	CCCCTC**I**G**I**AAAGCCTTCTTC	50.5	(7)

* GC-clump attached forward primer, *amoA*-1F-GC, was used in PCR-DGGE.
