# Supplementary material for: Comparison among amoA Primers Suited for Quantification and Diversity Analyses of Ammonia-Oxidizing Bacteria in Soil
Source: Microbes Environ. 2011 Nov 10;27(1):94–8. doi: 10.1264/jsme2.ME11230 (PMC4036032; doi:10.1264/jsme2.ME11230)
Supplement: Supplementary file 1 [file 27_94_s1.pdf]

**Legends of Figures**

Fig. S1: Amplification positions of *amoA* from *Nitrosospira multiformis* ATCC 25196 (accession number CP000103) for construction of *amoA* clones.

Fig. S2: DGGE band patterns of *amoA* fragments retrieved from gray lowland soil and andosol using the three primers at four different annealing temperatures.

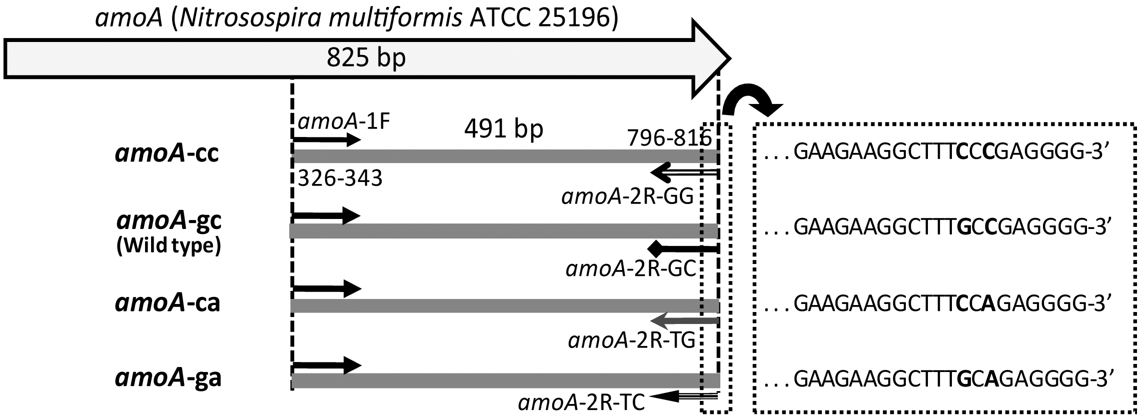


Shimomura et al. Figure S1

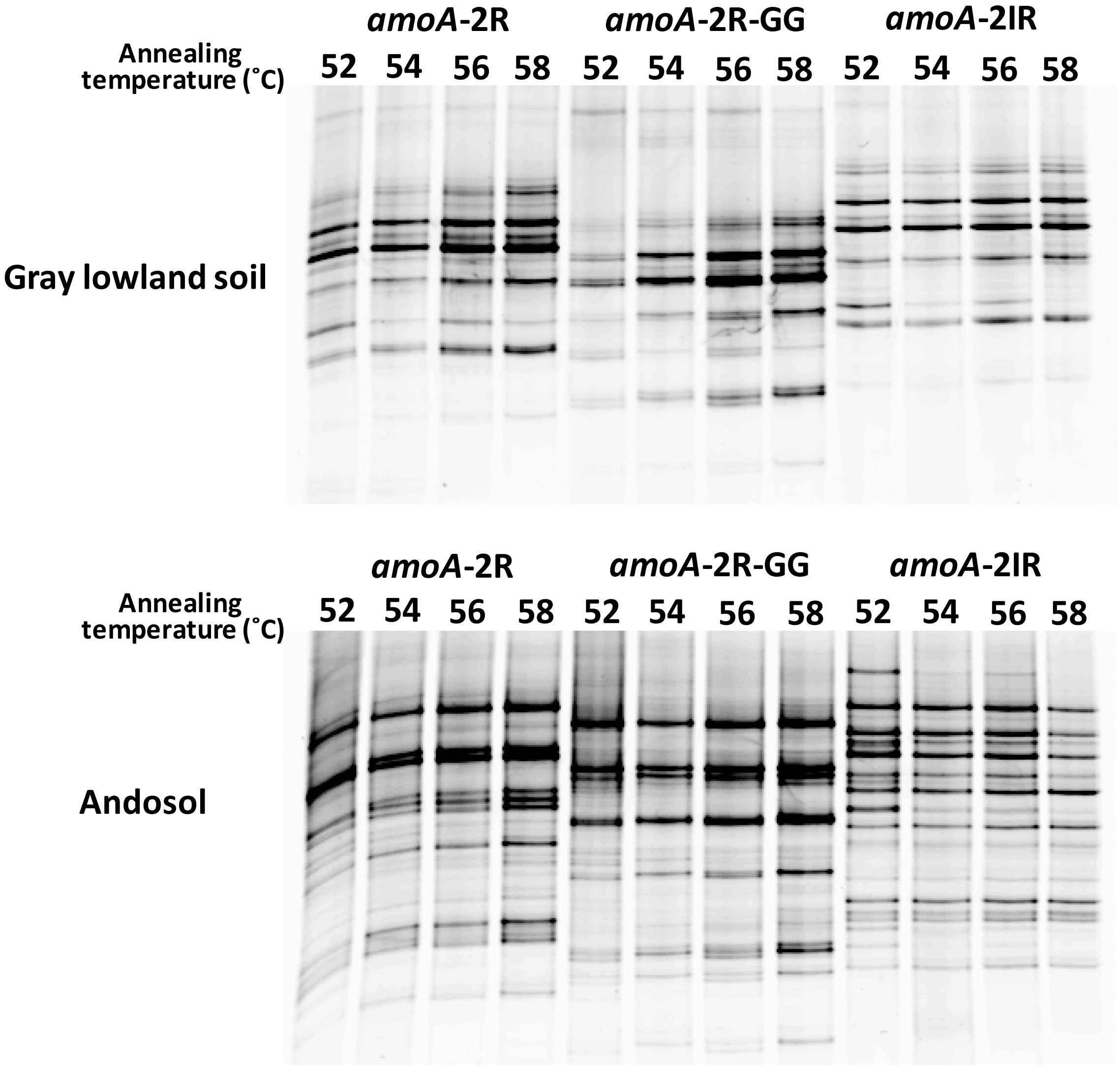


Shimomura et al. Figure S2

**Table S1.** PCR primers for the amplification of *amoA* gene fragments having complementary sequences with *amoA*-2R.

| Primer | | Sequence (5'-3') | Tm (ºC) | Referrence |
|---|---|---|---|---|
| Forward | *amoA*-1F | GGGGTTTCTACTGGTGGT | 46.5 | (26) |
| Reverse | *amoA*-2R-GG | CCCCTC**G**G**G**AAAGCCTTCTTC | 54.4 | (20) |
| | *amoA*-2R-GC | CCCCTC**G**G**C**AAAGCCTTCTTC | 54.4 | (20) |
| | *amoA*-2R-TG | CCCCTC**T**G**G**AAAGCCTTCTTC | 52.5 | (20) |
| | *amoA*-2R-TC | CCCCTC**T**G**C**AAAGCCTTCTTC | 52.5 | (20) |

**Table S2.** PCR conditions used in this study for the amplification of *amoA* gene fragments.

| Experiment | Template | Composition of reaction mixture | Thermocycling conditions |
|---|---|---|---|
| Construction of standard clones | *Nitrosospira multiformis* ATCC 25196 *amoA* | 25 μL of Premix Ex Taq™ Hot Start Version (Takara, Kyoto, Japan), 2 μL of 10 μM each primer (shown in Table S1), 1 μL of template (including $10^5$ *amoA* copies), and 20 μL of SDW. | 2 min at 94ºC, followed by 30 cycles at 94ºC for 30 s, at 54ºC for 30 s, and at 72ºC for 30 s. |
| Real-time PCR | DNA of standard clones or extracted soil DNA* | 10 μL of SYBR® Premix Ex Taq™ (Takara, Kyoto, Japan), 0.8 μl of 10 μM each primer (shown in Table 1), 0.2 μL of 20 mg/ml BSA, 0.4 μL of ROX Dye, 1.0 μL of template, and 6.8 μL of SDW. | 2 min at 94ºC, followed by 40 cycles at 94ºC for 30 s, at 54ºC for 30 s, and at 72ºC for 30 s. |
| PCR-DGGE | Extracted soil DNA | 25 μL of Premix Ex Taq™ Hot Start Version, 2 μL of 10 μM each primer (shown in Table 1), 1 μL of 20 mg/mL BSA, 1 μL of template (around 50 ng of soil DNA), and 19 μL of SDW. | 2 min at 94ºC, followed by 30 cycles at 94ºC for 60 s, at 52 to 58ºC by 2 ºC for 60 s, and at 72ºC for 60 s. |

Ten-fold dilution was used to measure *amoA* copy numbers in soil samples.

**Table S3.** Real-time PCR standard curves and calculated amplification efficiency of the three primer sets.

| Forward primer | Reverse primer | Template | Standard curve | Coefficient of determination | Amplification efficiency (%) |
|---|---|---|---|---|---|
| *amoA*-1F | *amoA*-2R | *amoA*-cc | y=−3.391+35.277 | $R^2$=0.9999 | 97.2 |
| | | *amoA*-gc | y=−3.551+35.49 | $R^2$=0.9999 | 91.3 |
| | | *amoA*-ca | y=−3.471+34.777 | $R^2$=1.0000 | 94.1 |
| | | *amoA*-ga | y=−3.382+31.804 | $R^2$=1.0000 | 97.6 |
| | *amoA*-2R-GG | *amoA*-cc | y=−3.4421x+36.115 | $R^2$=0.9999 | 95.2 |
| | | *amoA*-gc | y=−3.4031x+36.039 | $R^2$=0.9996 | 96.7 |
| | | *amoA*-ca | y=−3.5012x+36.433 | $R^2$=0.9999 | 93.0 |
| | | *amoA*-ga | y=−3.4455x+36.217 | $R^2$=0.9999 | 95.1 |
| | *amoA*-2IR | *amoA*-cc | y=−3.5105x+35.796 | $R^2$=0.9995 | 92.7 |
| | | *amoA*-gc | y=−3.4571x+35.663 | $R^2$=0.9991 | 94.7 |
| | | *amoA*-ca | y=−3.5559x+36.379 | $R^2$=0.9999 | 91.1 |
| | | *amoA*-ga | y=−3.4916x+36.097 | $R^2$=0.9998 | 93.4 |
